# Dissecting HealthBench: Disease Spectrum, Clinical Diversity, and Data Insights from Multi-Turn Clinical AI Evaluation Benchmark

**DOI:** 10.1007/s10916-025-02232-w

**Published:** 2025-07-28

**Authors:** Jialin Liu, Siru Liu

**Affiliations:** 1 Department of Otolaryngology-Head and Neck Surgery, West China Hospital, Sichuan University, Chengdu, China; 2 Department of Medical Informatics, West China Hospital, Sichuan University, Chengdu, China; 3 Department of Biomedical Informatics, Vanderbilt University Medical Center, 2525 West End Avenue, Suite 1475, Nashville, Nashville, TN, Tennessee 37203 USA

## Abstract

HealthBench is an open-source, large-scale benchmark consisting of 5,000 multi-turn clinical conversations evaluated against 48,562 criteria developed by clinicians. Recognized as a significant advancement in assessing realistic artificial intelligence (AI) models, HealthBench deserves further exploration. In this article, we systematically analyze the benchmark’s disease spectrum, diagnostic and therapeutic focuses, and demographic diversity. We evaluate its representativeness and strengths, as well as the essential limitations that AI researchers and clinicians should consider when using it for realistic model evaluations.

## Introduction

The rise of large language models (LLMs) in medicine demands benchmarks reflecting real-world clinical practice [1]. Traditional benchmarks like MedQA [2] (clinical knowledge recall) and PubMedQA [3] (biomedical literature) lack the conversational dynamics of clinical encounters. OpenAI’s HealthBench [4] addresses this gap, offering 5,000 multi-turn clinical conversations and 48,562 assessment criteria developed by 262 physicians across 60 countries, making it the most comprehensive conversational benchmark to date. However, its underlying clinical content—namely, the diseases represented, scenarios simulated, and patient demographics captured—has not been systematically characterized. In this study, we quantitatively and qualitatively analyze HealthBench’s disease spectrum, conversational complexity, and data structure to provide actionable insights for both the medical and artificial intelligence (AI) communities.

First, we determined the language of each clinical conversation and translated non-English content into English. Subsequently, we used LLMs with Azure AI Translator and Azure Text Analytics for Health to extract diagnoses, symptoms, signs, treatments, examinations, condition qualifiers, and medications from each query. To validate the accuracy of this automated extraction process, 100 conversations were randomly sampled from the total set of 5,000 and manually reviewed to verify the accuracy of the extracted health information.

### Demographic and Linguistic Profile of HealthBench

Of the 5,000 clinical conversations in HealthBench, 1,030 mention patient age: 368 (35.7%) for ages 0–17, 236 (22.9%) for 18–35, 279 (27.1%) for 36–65, and 147 (14.3%) for over 65. Gender is specified in 332 conversations: 194 (58.4%) female, 135 (40.7%) male, and 3 (0.9%) other gender identities. Most conversations are in English (4,248; 85.0%), followed by Spanish (192; 3.8%) and Portuguese (173; 3.5%), with 387 (7.7%) across 29 other languages.

### Dataset Coverage

The HealthBench dataset’s 5,000 multi-turn clinical conversations, annotated with Systematized Nomenclature of Medicine - Clinical Terms (SNOMED CT), include 4,975 (99.5%) with at least one medical entity, averaging 19.2 entities across 36 categories. The top five categories—symptom or sign (23,024; 23.9%), treatment name (12,457; 12.9%), diagnosis (11,621; 12.1%), examination name (8,132; 8.5%), condition qualifier (5,526; 5.7%)—total 60,760 mentions (63.1%). Only 25 conversations (0.5%) lack entities (e.g., toddler bedtime, raw milk safety). A manual review of 100 samples found no missed entities, suggesting high accuracy but requiring broader validation.

### Conversation Complexity

The HealthBench dataset comprises 5,000 multi-turn clinical conversations, with a mean word count of 107.5 (Q1: 18.0; Q3: 154.0), reflecting diverse conversation lengths and information density. Each conversation contains an average of 5.1 distinct medical relations. High-complexity conversations, defined as those exceeding the 90th percentile on a composite metric integrating medical entity count, relation density, and diagnostic variety, constitute 499 cases (10.0%). Furthermore, 1,260 conversations (25.2%) involve multiple medical specialties, facilitating evaluation of cross-disciplinary reasoning. Entity annotations have a mean confidence score of 0.904, which indicates robust reliability.

### Disease Distribution

**Fig. 1:**
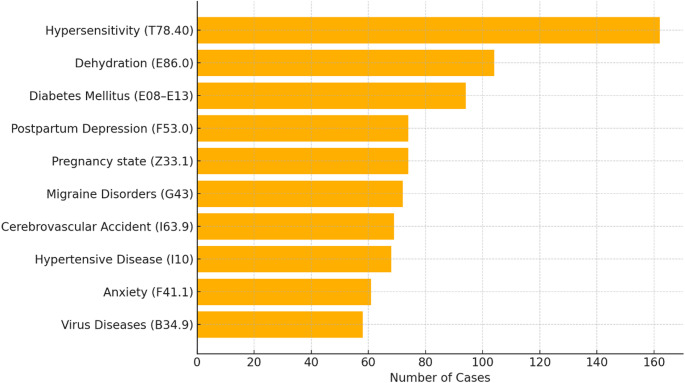
Top 10 most frequently mentioned diseases in the HealthBench

Among the conversations, 2,270 (45.4%) explicitly reference one or more diseases, encompassing 674 unique diseases. Across all conversations, these diseases are mentioned a total of 3,837 times. The dataset covers all 21 International Classification of Diseases, Tenth Revision (ICD-10) disease chapters, except the U codes for special purposes, and spans 26 primary clinical specialties. The ten most frequently mentioned diseases are hypersensitivity (ICD-10 T78.40, 162 mentions), dehydration (E86.0, 104), diabetes mellitus (E08–E13, 94), postpartum depression (F53.0, 74), pregnancy (Z33.1, 74), migraine disorders (G43, 72), cerebrovascular accident (I63.9, 69), hypertensive disease (I10, 68), anxiety (F41.1, 61), and viral diseases (B34.9, 58) (Fig. 1). Additionally, rare diseases were defined using the Orphanet nomenclature (prevalence < 1/2,000) and confirmed by physician review. HealthBench thus includes 57 rare diseases—8.5% of all diseases and 176 mentions (4.7% of total disease mentions)—among them amyotrophic lateral sclerosis (ORPHA:803) and retinitis pigmentosa (ORPHA:791).

### Therapeutic Interventions

#### Medications

In the HealthBench dataset of 5,000 multi-turn clinical conversations, 42 medication categories, based on RxNorm classification, are referenced in 292 mentions. The five most frequently referenced medication classes according to Anatomical Therapeutic Chemical Classification System (ATC) level-2 codes—analgesics (N02, 51 mentions), antidepressive agents (N06A, 41), vitamins (A11, 39), adrenergic beta-antagonists (C07, 32), and diuretics (C03, 22), with selective serotonin reuptake inhibitors (N06AB, 22) sharing the fifth rank—account for 70.9% (207/292) of all mentions. Of these, 33 categories (78.6%) are listed in the World Health Organization’s Essential Medicines List (EML, version 23, 2023) [5]. Additionally, 521 distinct medications are identified, totaling 1,429 mentions. The most common medications are ibuprofen (61 mentions), acetaminophen (47 mentions), amoxicillin (33 mentions), epinephrine (24 mentions), warfarin (22 mentions), and vitamin D (22 mentions). These span acute and chronic conditions across specialties, including pediatrics, geriatrics, mental health, oncology, and infectious diseases, supporting comprehensive clinical evaluation.

#### Surgical and Interventional Procedures

The HealthBench dataset comprises 5,000 multi-turn clinical conversations, in which a total of 492 mentions of 110 unique surgical and interventional procedures are identified. The most frequent procedures—interventional procedure (SNOMED CT: 71388002, 100 mentions), operative surgical procedure (257556004, 87), surgical reduction (289928003, 40), removal technique (118292001, 26), intubation (52765003, 11), dental bridge fixation (234799009, 11), and cesarean Sects. (11466000, 11)—account for 58.1% (286/492) of mentions. These procedures span 24 clinical specialties, including general surgery, orthopedics, obstetrics, and dentistry, facilitating assessment of model performance across diverse surgical contexts.

#### Clinical Utility and Positioning

Our analysis of HealthBench reveals that its disease spectrum does not mirror clinical epidemiology but instead captures a unique, “query-driven” distribution of health topics. This characteristic is precisely what makes it a valuable tool for evaluating patient-facing conversational AI.

The frequency of specific conditions in HealthBench provides key clinical insights. The prominence of acute issues like hypersensitivity and dehydration suggests the benchmark is well-suited to evaluate an AI’s ability to handle urgent, user-initiated queries that require rapid assessment. The inclusion of chronic diseases like diabetes and hypertension highlights its utility in assessing AI-driven tools for patient education and self-management. Furthermore, the presence of sensitive topics like postpartum depression and anxiety underscores the benchmark’s capacity to test AI performance in mental health, a domain where users may prefer the anonymity of a conversational agent.

Compared to other benchmarks, HealthBench occupies a unique position. Its content distribution is not driven by a medical curriculum, as in MedQA, or by research trends, as in PubMedQA. While MedQA simulates a “doctor’s brain” of textbook knowledge and PubMedQA a “researcher’s brain” of scientific literature, HealthBench simulates a “patient’s voice.” It reflects the health concerns that are top-of-mind for the public, including lifestyle topics like pregnancy, which are common queries but not typically framed as “exam questions”. This distinction makes it an indispensable resource for evaluating an AI’s ability to apply medical knowledge in the nuanced, unpredictable context of a real-world conversation.

#### Limitations and Future Directions

While HealthBench represents a significant advancement in medical AI benchmarking with realistic multi-turn conversations, several limitations should be considered. First, most conversations are synthetically generated rather than de-identified real-world transcripts, which may omit idiomatic phrasing, topic shifts, and unanticipated patient concerns. Second, rare diseases remain under-represented (57/674 disease; 176/3,837 mentions), constraining evaluation in low-prevalence or complex scenarios. Third, the focus on short conversations precludes assessment of longitudinal workflows and multidisciplinary handovers, limiting insights into AI’s impact across the complete care continuum. Finally, entity extraction relied on automated tools with manual validation of only 100 conversations (2%), which may not fully capture nuanced language use or error modes; a broader validation is needed to quantify precision and recall comprehensively.

To enhance HealthBench’s clinical value and generalizability, we propose a multi-pronged strategy. This strategy focuses on three key areas: (1) diversifying data content and representation, (2) increasing clinical realism and complexity, and (3) establishing robust quality governance. First, the targeted enrichment of the dataset’s content is paramount for equity and utility. While the current dataset includes some rare diseases, a more strategic approach is needed. Collaborate with institutions like the National Organization for Rare Disorders to curate more than 500 additional rare disease conversations, focusing on conditions with a prevalence < 1/100,000. Future work should prioritize targeted enrichment of high-acuity clinical scenarios that are currently underrepresented, such as rare pediatric conditions (e.g., inborn errors of metabolism), complex autoimmune disorders, and atypical disease presentations. A concrete strategy would be to partner with specialized clinical centers and patient advocacy groups to generate validated, high-fidelity conversational data. Similarly, a systematic expansion to increase the representation of minority and marginalized populations is a critical prerequisite for evaluating and promoting health equity. Second, the benchmark must evolve better to reflect the complexity and continuity of actual healthcare. The current reliance on synthesized conversations limits HealthBench’s ability to capture the unpredictability of real-world interactions. Incorporating de-identified real-world patient–clinician transcripts is essential. This will test model resilience to idiomatic phrasing, incomplete clinical details, and subtle nuances in communication. Furthermore, clinical practice is not a series of isolated encounters. Extending HealthBench to include longitudinal and multidisciplinary scenarios—such as follow-up visits, specialist handoffs, and documentation of patient outcomes—is crucial for evaluating an AI’s impact across the entire care continuum, not just in a single snapshot. Finally, ensuring the long-term relevance and reliability of HealthBench requires a framework for continuous quality assurance and stakeholder-driven governance. This involves establishing transparent processes for external validation of both the dataset’s content and the evaluation rubrics. Creating a platform for stakeholder-driven curation would allow the benchmark to be continuously updated and expanded as medical knowledge evolves, ensuring its ongoing relevance and trustworthiness for AI and clinical research stakeholders.

## Conclusion

HealthBench marks a significant advance in the realistic evaluation of medical AI. Our analysis demonstrates its value as a robust foundation for clinically relevant model assessment, while also highlighting key areas for future improvement. Specifically, HealthBench can support critical clinical validations including diagnostic accuracy, therapeutic decision-making, medication management, patient education, and chronic disease follow-up, thereby ensuring AI systems meet real-world clinical standards. As artificial intelligence continues to transform healthcare, ongoing efforts to refine and expand evaluation datasets like HealthBench will be critical to ensuring that future systems are safe, equitable, and truly beneficial in clinical practice.

## Data Availability

No datasets were generated or analysed during the current study.

## Abbreviations

LLM: Large language models
AI: Artificial intelligence
SNOMED-CT: Systematized nomenclature of medicine - clinical terms
ICD-10: The international classification of diseases, tenth revision
ATC: Anatomical therapeutic chemical classification system
RxNorm: Normalized names for clinical drugs
